# Learners as Teachers: Development of a Novel Faculty Development Curriculum Utilizing Junior Faculty as Primary Authors

**DOI:** 10.12688/mep.21371.1

**Published:** 2025-11-25

**Authors:** Heather A Brown, Stanley Hassinger

**Affiliations:** 1 Emergency Medicine, Prisma Health Richland Hospital, Columbia, South Carolina, 29203, USA

**Keywords:** faculty development, curriculum development, graduate medical education, junior faculty, professional development

## Abstract

**Background:**

Academic faculty development varies in scope and utility. Timely, specialty-specific faculty development helps support junior faculty and should be incorporated early in their academic careers. Junior faculty likely have the most to gain from faculty development but are rarely included in its creation. This study aimed to create and deploy an effective faculty development curriculum for junior emergency medicine (EM) faculty through experiential learning by using junior faculty as primary authors.

**Methods:**

Eight senior faculty developed a list of high-yield topics. Seven junior faculty were assigned as primary authors for each of the topics according to interest. A senior faculty considered an expert in the area was assigned as a mentor. Topics were presented at a peer review session with all participants present. Following the session, participants took an electronic survey consisting primarily of 5 point Likert scale questions with an 86% response rate.

**Results:**

Survey responses highlight faculty development as an academic gap and strongly support the curriculum’s importance and effectiveness. All responding junior faculty and the majority of senior faculty strongly agreed that creation of the modules and participation in the peer review process improved their knowledge (median of 5 for both questions). All respondents strongly agreed the topics covered were important and should be mandatory for all new academic faculty.

**Conclusions:**

Using junior faculty as primary authors to create a specialty and institutional specific faculty development curriculum is an effective approach to create and deliver a curriculum. This method is cost-effective, well-received and sustainable as it builds on and strengthens pre-existing resources and relationships. This method also succeeded in creating a community of practice dedicated to sharing knowledge and skills around medical education. Given its generalizable framework, this model of curriculum development could likely be deployed in any graduate medical program regardless of specialty.

## Introduction

Faculty development is an important component of ensuring the delivery of high-quality graduate medical education (GME). Many GME accreditation bodies require ongoing faculty development as a contingency for program accreditation ^1, 2^. Faculty development opportunities vary widely in scope, methods of delivery, and perceived utility. There is no authoritative list of faculty development topics for GME programs to follow and recent studies suggest that up to half of U.S. emergency medicine (EM) faculty may be unsatisfied with the faculty development they receive ^3^.

While many topics are pertinent across medical specialties, the setting in which care is delivered, and in which resident education occurs, can impact which faculty development content is most relevant. Many faculty rely on their national specialty organizations to provide faculty development experiences ^4– 6^. Most of these opportunities are off-site and in-person, which may limit group participation within a single department, and are often costly. These programs rarely provide mentorship opportunities, which have been identified among the career development needs of young faculty ^4^. When larger scale faculty development opportunities do incorporate mentorship, it is often difficult to foster a close mentor-mentee relationship given geographic distances. In contrast, local institution based faculty development like the program we describe here has no such limitation and effectively promotes close mentorship relationships that may even extend beyond academic mentorship. Beyond formal mentorship relationships, participation in communities of practice (CoP), defined as a group of people “engaged in a process of collective learning in a specific domain”, can improve faculty teaching, leadership and research skills through less formal avenues of faculty development ^7– 10^.

While creating a specialty and site-specific curriculum is likely most beneficial to learners, the process can be laborious and daunting. Additionally, junior faculty likely have the most to gain from faculty development but are rarely included in its creation. Although senior faculty have more expertise in most topics pertinent to faculty development, junior faculty may have better insight into their own educational needs and may benefit more from a “learning by doing” approach to faculty development. Here we test that hypothesis through the creation and deployment of a faculty development curriculum for EM junior faculty within a single EM residency program by utilizing junior faculty as primary authors and pairing them with a senior faculty mentor within the department. The program sought to expand junior faculty’s expertise in an area of academic interest and build a community of practice.

## Methods

Eight senior faculty developed a list of high-yield topics for junior faculty and the most useful were selected by consensus using a nominal group technique ( Table 1). All junior faculty working primarily at the academic center were invited to participate and all seven agreed. Senior faculty were selected to participate based on their areas of expertise and years of experience to provide a balanced group. All senior faculty invited to participate accepted. To promote independent learning and collaboration, junior faculty were each assigned as primary authors for one topic according to their interest. Each junior faculty was assigned a senior faculty mentor considered an expert in the topic area. Each faculty pair had six months to create a module to be presented in didactic lecture format. A shared electronic document repository was created and participants were encouraged to add relevant documents to the repository including relevant journal articles, tip sheets, and other useful documents. Topics were presented during a peer review session with all participants present. Completed electronic presentations were also added to the electronic document repository for future reference.

**Table 1.** Curriculum Topics and Associated Deliverables.

Topic	Deliverables
Navigating the IRB and protocol development	□ Complete Citi Training □ Register for IRB account □ Request redcap account
Writing and submitting a scientific manuscript	□ Identify relevant journals for research topic
Administrative skills	□ Quality improvement project signup
ACGME faculty requirements and tenure track criteria	□ Review CV □ Apply for academic appointment
Leadership	□ Hospital committee sign up
Teaching outside the box	□ Lecture development
Effective Mentorship/Giving effective feedback	□ Identify mentor □ Write goals for mentorship
Additional topics to add, as recommended by the participating faculty and fellows: - Journal peer review and editing - Grant writing - Quality improvement - How to give a great presentation	

At the conclusion of the peer review session, participants were asked to complete a 12-question electronic survey primarily consisting of 5-point Likert scale questions assessing perceived utility of the training and the module development process. Responses were tallied and reported as percentages. Survey data was collected anonymously as routine evaluation of the educational session and retrospectively submitted for review by the Prisma Health IRB [2311472-1] with a designation of exempt status. Due to the anonymous and retrospective nature of the survey data, informed consent was waived. However, all participants did verbally consent to have their anonymously collected survey results published. All activities took place over six months with the peer review session and survey completion in February of 2024. This curriculum and survey results were first presented at the annual Council of Residency Directors in Emergency Medicine conference in Seattle, Washington on March 3, 2025 ^11^.

## Results

Six junior faculty and six senior faculty completed the survey for an 80% response rate ( Table 2). Only 33% of respondents agreed that they had received sufficient formal education on the faculty development topics before the peer review session. All respondents strongly agreed that the topics were important for fellows and faculty to understand and that the completion of the curriculum should be mandatory for all new academic faculty. All respondents agreed that the curriculum should be mandatory for all future fellows and all core faculty. All respondents agreed, and all responding junior faculty strongly agreed, that creation of the modules and participation in the peer review process improved their knowledge. No respondents suggested removing any of the topics presented from the curriculum. Several additional topics were suggested and are included in Table 1. Additional comments included a suggestion that the curriculum would have utility for resident physicians as well, and a suggestion that starting off with a faculty needs assessment may be helpful.

**Table 2.** Participant Survey Results.

	Participant Survey Results				
	The topics covered were important for fellows and new faculty to understand				
	Strongly agree	Agree	Neutral	Disagree	Strongly Disagree
**Junior Faculty**	100% (6/6)	0%	0%	0%	0%
**Senior Faculty**	100% (6/6)	0%	0%	0%	0%
	Creating the assigned module greatly increased my knowledge of the topic				
	Strongly agree	Agree	Neutral	Disagree	Strongly Disagree
**Junior Faculty**	100% (6/6)	0%	0%	0%	0%
**Senior Faculty**	67% (4/6)	33% (2/6)	0%	0%	0%
	Participating in the peer review process greatly increased my knowledge of the topic				
	Strongly agree	Agree	Neutral	Disagree	Strongly Disagree
**Junior Faculty**	100% (6/6)	0%	0%	0%	0%
**Senior Faculty**	83% (5/6)	17% (1/6)	0%	0%	0%
	The curriculum should be mandatory for all future fellows				
	Strongly agree	Agree	Neutral	Disagree	Strongly Disagree
**Junior Faculty**	83% (5/6)	17% (1/6)	0%	0%	0%
**Senior Faculty**	83% (5/6)	17% (1/6)	0%	0%	0%
	The curriculum should be mandatory for all future new academic faculty				
	Strongly agree	Agree	Neutral	Disagree	Strongly Disagree
**Junior Faculty**	100% (6/6)	0%	0%	0%	0%
**Senior Faculty**	100% (6/6)	0%	0%	0%	0%
	**I have received sufficient formal education on these topics before this activity**				
	Strongly agree	Agree	Neutral	Disagree	Strongly Disagree
**Junior Faculty**	33% (2/6)	0%	17% (1/6)	17% (1/6	33% (2/6)
**Senior Faculty**	17% (1/6)	0%	50% (3/6)	33% (2/6)	0%

## Discussion

This curriculum development process is cost-effective and logistically less challenging compared to sending junior faculty to national conferences or bootcamps. In addition, because its development is site-specific, the curriculum addresses institutional specific processes and challenges. This approach to curriculum development is unique in several ways which are particularly beneficial to participants.

### Junior faculty as teachers

Faculty development programs rarely involve junior faculty in the planning or delivery of the curriculum. This represents a missed opportunity given the highly positive feedback from all junior faculty regarding the knowledge they gained by creating the modules. Junior faculty chose their topics with each selecting an area important to their career goals and allowing them to create a new niche area of expertise for themselves. To our knowledge, no other published graduate medical faculty development curriculum has used junior faculty as primary authors. While this curriculum was developed by and for EM faculty, the process of curriculum development could likely be replicated with similar results within any GME program regardless of specialty and other cadres of medical professionals as well.

### Peer review session

The peer review session allowed the full content of the curriculum to be delivered to all junior faculty in the department at once, effectively training the target audience in a single day. This approach to curriculum development could be particularly useful for newer programs looking to create faculty development opportunities quickly as the entire program was developed and deployed over a six month period. The peer review session served as a de facto needs assessment with participants identifying additional areas of interest during the session which were subsequently incorporated into the curriculum over time.

### Mentorship

The process we describe has the added benefit of creating new mentorship relationships within the department in an organic fashion through the collaboration of module development. This approach to curriculum development successfully established a CoP within our department dedicated to sharing educational knowledge and skills. The peer review session in particular fostered faculty cohesiveness and enthusiasm around the program with a majority of core faculty participating including the department chair and program director. Since curriculum deployment, new educational strategies are being employed by faculty and there is an increased emphasis on academic promotion. The curriculum is now being delivered to fellows and new academic faculty in quarterly modules, with significant ongoing participation from the original participants.

### Limitations

This pilot study included a small sample size at a single institution. Future work could expand the implementation to other GME programs and additional sites, providing the opportunity to collect broader feedback and outcome data. Involving junior faculty in a needs assessment in the initial stages of curriculum planning may have created an even more useful or targeted curriculum and should be considered if the process is replicated. Changes in faculty knowledge and behavior were not directly measured. A future evaluation of how the education and resources utilized over a longer period of time could further inform similar curricula.

## Conclusion

Given its generalizable framework, this model of curriculum development could likely be deployed in any graduate medical program regardless of specialty as well as other cadres of medical professionals. This approach to faculty development is cost-effective, well-received and sustainable as it builds on and strengthens pre-existing resources and relationships. Programs interested in utilizing this approach should consider including junior faculty in the initial planning stages either through a formal needs assessment or inclusion in the process of topic selection. Additionally, the peer review session presented here was in-person which we believe was crucial to the success of the program in garnering enthusiasm and creating a CoP. This is supported by previous studies which identify in-person interactions as a key facilitator in developing CoP ^8^. We encourage programs to consider this model and offer this work as an example to build on. 

## Ethics approval

The study was review by the Prisma Health IRB [2311472-1] and approved with a designation of exempt status.

## Data Availability

Survey results are presented in this paper in aggregated form in Table 2. Supplementary materials including survey questions, raw survey data and STROBE checklist are available as extended data from *Learners as Teachers: Development of a Novel Faculty Development Curriculum Utilizing Junior Faculty as Primary Authors* on Open Science Framework under the terms of the Creative Common Zero (CC0) license. Openly available at https://doi.org/10.17605/OSF.IO/MU5FT ^12^.
