# Supplementary material for: Learners as Teachers: Development of a Novel Faculty Development Curriculum Utilizing Junior Faculty as Primary Authors
Source: MedEdPublish (2016). 2025 Nov 25;15:274. [Version 1] doi: 10.12688/mep.21371.1 (PMC12848336; doi:10.12688/mep.21371.1)
Supplement: Peer review history

# Reviewer report: Reviewer response for version 1

**DOI:** 10.21956/mep.22888.r45107

**Published:** 2026-01-28

**Authors:** Nasibah Azme

**Affiliations:** 1 Universiti Teknologi MARA (UiTM), Sungai Buloh, Malaysia

**Overall:**

 The structure of this manuscript presents a well-conceived and innovative model of faculty development that utilizes junior faculty as the key curriculum developers, with the assistance of senior faculty mentors in the EM residency program.

 Nevertheless, one or two aspects still need further explanation as detailed below. These points should be addressed to increase the rigor of the study.

**Introduction:**

 1. "There is no authoritative list of faculty development topics for GME programs to follow and recent studies suggest that up to half of U.S. emergency medicine (EM) faculty may be unsatisfied with the faculty development they receive ^3^" 

 Recent studies suggest that there is more than one source, yet one source is mentioned. The authors can either rephrase to portray a single study or provide additional supporting sources.

 2. "junior faculty may have better insight into their own educational needs and may benefit more from a “learning by doing” approach to faculty development."

 Learning by doing here is an assumption; please incorporate more evidence on this, or at least cite any framework for this, either conceptual or theoretical.

**Methods:**

 "Eight senior faculty developed a list of high-yield topics for junior faculty and the most useful were selected by consensus using a nominal group technique"

 Please clearly define the criteria used to classify senior and junior faculty. Clarification on how seniority and junior status were determined is important (e.g how many years of service etc), as this may influence the study outcomes.

**Results:**

 Please describe more about the instrument, whether it is validated, and any adaptation to the instrument

**Discussion:**

 I'm not sure if there is a limitation for the references for this type of manuscript; i.e brief report. But, the discussion should be supported by relevant prior literature, ideally with one or two references per major claim, even if drawn from different disciplines/field.

Have any limitations of the research been acknowledged?

Yes

Is the study design appropriate and does the work have academic merit?

Yes

Is the work clearly and accurately presented and does it cite the current literature?

Partly

If applicable, is the statistical analysis and its interpretation appropriate?

Yes

Are all the source data underlying the results available to ensure full reproducibility?

Yes

Are the conclusions drawn adequately supported by the results?

Yes

Are sufficient details of methods and analysis provided to allow replication by others?

Partly

Reviewer Expertise:

Medical Education, Innovative Teaching, Faculty Development, Technology in Higher Education

I confirm that I have read this submission and believe that I have an appropriate level of expertise to confirm that it is of an acceptable scientific standard, however I have significant reservations, as outlined above.

# Reviewer report: Reviewer response for version 1

**DOI:** 10.21956/mep.22888.r45108

**Published:** 2026-01-27

**Authors:** Sherri Rudinsky

**Affiliations:** 1 Uniformed Services University of the Health Sciences, Bethesda, Maryland, USA

This is a pragmatic brief report that addresses a significant gap in academic faculty development that exists in many programs. The authors describe an innovative "learning by doing" approach where junior faculty serve as primary authors of a specialty-specific faculty development curriculum. The study demonstrates that this model is feasible and well-received within a single Emergency Medicine department. While the results are encouraging, the manuscript would be strengthened by providing a stronger theoretical foundation and increased methodological transparency, particularly, in regards to mentor selection and survey development.

 Introduction:  The claim that junior faculty have better insight into their own needs through "learning by doing" is compelling. To strengthen this argument, consider more explicitly clarifying which pedagogical theories support this premise (e.g. self-directed learning, experiential learning). To better situate the work within the current scholarship landscape, consider including one or two more recent references (2024–2025) regarding Communities of Practice or experiential faculty development.

 Methods: The curriculum topics were selected by senior faculty using a nominal group technique. Given the learner-centered nature of the study, please explain why a senior-led approach was chosen over a mixed-needs assessment with junior faculty. Was the topic selection informed at all by a review of relevant literature or national organizations? 

 As significant importance is placed on the experiential nature of the learning experience, an explanation as to why the curriculum was limited to a group session of didactic lectures would be helpful. A “curriculum” and “module” implies more than just a series of lectures, and other components could be better defined to assist with reproducibility. 

 It would be helpful to provide more detail on how mentor "expertise" was operationalized (e.g. publication record, years of experience, informal reputation) and whether the mentoring interactions followed a specific timeline or structure. This would aid in validity (defining expertise) as well as reproducibility. While some things can be inferred, it would also be helpful to contextualize the setting by providing some description of the program - academic, UME, GME, fellowships etc, size of program (e.g. 8 out of 40 faculty volunteered or 8 out of 10?). 

 The statement “all eligible junior faculty participated” opens up to some potential bias regarding the selection of participants. This unanimous participation may suggest a group that was already highly motivated or academically inclined or a perceived inability to decline. What was the criteria/definition for “junior” faculty?

 Please describe how the 12-item electronic survey was developed. Clarify if the tool was locally designed, expert-reviewed, or adapted from existing validated instruments. The questions appear geared toward the junior faculty experience. This may not be the “right” survey for the faculty - a potential exists to gain useful information about the faculty experience as mentors (what was gained/learned about serving as mentor - a skill in and of itself?) The results suggest some additional data gathering beyond the 12-questions. It would be helpful to better define this (e.g. open ended questions, formal after action, informal discussions etc.)

 Results: Given the small sample size and participants being known within the program, I was surprised at the response rate (being less than 100%). Was there any particular reason as to why? 

 These results showed uniformly high levels of agreement. Given the small sample size, the findings should be interpreted cautiously. It would be helpful to discuss any safeguards used (anonymity or triangulation with qualitative feedback), to reduce the risk of overinterpretation.

 Discussion: The peer review session is a central feature of the model. More explicitly connecting this session to peer-learning or communities of practice frameworks would show the design was theoretically informed rather than just pragmatic.

 Mentorship is a learned skill and can/should be developed amongst faculty. The mentorship impact of this curriculum was inferred, but I would consider noting how future iterations could use explicit outcome indicators to measure this impact directly.

 Faculty development as a facilitator for academic promotion was mentioned. It would be interesting to know if this program translated into any extramural scholarly dissemination for the junior faculty who participated.

 The limitations section should be expanded to acknowledge potential bias in the inherent power dynamics between junior faculty and senior mentors serving also as evaluators.

 Conclusion: There were multiple opportunities to be more explicit in the description of the curricular process that would allow reproducibility. The claim that this model is generalizable to any graduate medical program is reasonable but should be softened to frame it as transferable in principle rather than universally applicable.

 Disclaimer: The opinions and assertions expressed herein are those of the author(s) and do not reflect the official policy or position of the Uniformed Services University of the Health Sciences or the Department of War.

Have any limitations of the research been acknowledged?

Yes

Is the study design appropriate and does the work have academic merit?

Partly

Is the work clearly and accurately presented and does it cite the current literature?

Yes

If applicable, is the statistical analysis and its interpretation appropriate?

Yes

Are all the source data underlying the results available to ensure full reproducibility?

Partly

Are the conclusions drawn adequately supported by the results?

Partly

Are sufficient details of methods and analysis provided to allow replication by others?

Partly

Reviewer Expertise:

Emergency Medicine, medical education (UME, GME), health professions education

I confirm that I have read this submission and believe that I have an appropriate level of expertise to confirm that it is of an acceptable scientific standard, however I have significant reservations, as outlined above.

# Reviewer report: Reviewer response for version 1

**DOI:** 10.21956/mep.22888.r45110

**Published:** 2026-01-27

**Authors:** YC Nalini

**Affiliations:** 1 PHYSIOLOGY, Sri Manakula Vinayagar Medical college and Hospital, Pondicherry, Puducherry, India

This manuscript presents a **well-conceived, pragmatic, and innovative faculty development model** that leverages junior faculty as primary curriculum developers, paired with senior faculty mentors, within an Emergency Medicine (EM) residency program. However, providing justification on these points will help the reader comprehend better.

 1) Elaborating details of survey tool (adapted, self-designed) would add rigour to the paper.

 2) Providing details regarding validation process. (face validity, content validity)

Have any limitations of the research been acknowledged?

Yes

Is the study design appropriate and does the work have academic merit?

Yes

Is the work clearly and accurately presented and does it cite the current literature?

Yes

If applicable, is the statistical analysis and its interpretation appropriate?

Partly

Are all the source data underlying the results available to ensure full reproducibility?

Yes

Are the conclusions drawn adequately supported by the results?

Yes

Are sufficient details of methods and analysis provided to allow replication by others?

Partly

Reviewer Expertise:

medical education, simulation, physiology

I confirm that I have read this submission and believe that I have an appropriate level of expertise to confirm that it is of an acceptable scientific standard.

# Reviewer report: Reviewer response for version 1

**DOI:** 10.21956/mep.22888.r44651

**Published:** 2025-12-30

**Authors:** Kallia Katsampoxaki-Hodgetts

**Affiliations:** 1 Technical University of Crete, Chania, Crete Region, Greece

**This is a strong, timely, and well-executed brief report that makes a meaningful contribution to the faculty development literature, particularly within graduate medical education. The manuscript is clearly written, the intervention is pragmatic and innovative, and the authors convincingly demonstrate feasibility, acceptability, and perceived value. The emphasis on junior faculty as curriculum authors, paired with structured peer review and mentorship, represents a refreshing departure from more traditional, top-down faculty development models.**

**My comments herein are offered in the spirit of enhancing conceptual clarity, methodological transparency, and scholarly positioning, rather than addressing fundamental flaws. Addressing these points would further strengthen the paper’s impact, credibility, and transferability.**

**In the Introduction, the authors state that “junior faculty may have better insight into their own educational needs and may benefit more from a ‘learning by doing’ approach to faculty development.” This is a compelling claim and one that aligns with contemporary adult learning theory. I believe it would strengthen the manuscript to clarify which learning theories or pedagogical approaches or practices support the premise that junior faculty possess superior insight into their own learning needs (e.g., self-directed learning, experiential learning, communities of practice, ergative knowledge processes in Learning-by-design).**

**In the Methods, the authors note that “eight senior faculty developed a list of high-yield topics… selected by consensus using a nominal group technique.” Given the learner-centred ethos of the curriculum, this design choice warrants further reflection. Specifically, it would be helpful for the authors to explain why a senior-faculty-led nominal group technique was preferred over a junior-faculty or mixed needs assessment, particularly when junior faculty are positioned as the primary beneficiaries and co-constructors of the curriculum. This is especially relevant given that senior faculty may not necessarily have formal pedagogical training, as is common in clinical academia.**

**The manuscript also states that each junior faculty member was paired with “a senior faculty mentor considered an expert in the topic area.” This mentoring structure is clearly central to the intervention’s success. However, readers would benefit from greater clarity regarding how expertise was defined and operationalised. Were mentors selected based on publication record, leadership roles, years of experience, prior mentoring experience, or informal reputation? Additionally, it is unclear whether mentoring interactions were left entirely emergent or whether any shared expectations, timelines, or minimal structures were in place. Addressing this in detail would help readers assess comparability of learning conditions across participants and better understand how the model might be replicated elsewhere.**

**In the Methods, all eligible junior faculty were invited and all agreed to participate. While this speaks to the perceived relevance of the initiative, it also raises methodological questions. In fact, how do the authors account for potential selection effects associated with voluntary and unanimous participation? For example, participants may already have been highly motivated, academically inclined, or positively disposed toward faculty development. A brief acknowledgement of how this may influence objectivity, response patterns, and transferability would strengthen the paper’s critical reflexivity.**

**In both the Methods and Discussion, the peer review session is presented as a central design feature, functioning as a delivery mechanism, a collaborative learning space, and a de facto needs assessment. While its value is evident, the pedagogical rationale remains largely implicit. It would strengthen the manuscript to more explicitly connect the peer review session to contemporary peer-learning, experiential faculty development, or community-of-practice frameworks, particularly those published in the last five years. Doing so would help position this design choice not only as pragmatic but also as theoretically informed.**

**The Methods describe a 12-item Likert-scale survey assessing perceived utility and learning. While this is appropriate for a pilot study, additional clarification would enhance rigor. In particular, readers may ask how the survey was developed (e.g., adapted from existing instruments, locally designed, expert reviewed) and what evidence supports its content validity. Furthermore, since outcomes focus on satisfaction and perceived knowledge gains, it would be useful to acknowledge the limits of such measures and clarify what claims the authors believe are and are not supported by these data.**

**In the Results, agreement levels are uniformly high across junior and senior faculty. While encouraging, such results can be difficult to interpret in small samples. The manuscript would benefit from a brief discussion of how the authors interpret these findings cautiously, and whether any methodological safeguards (e.g., anonymity, item phrasing, triangulation with qualitative feedback) were considered to reduce the risk of overinterpretation.**

**In the Discussion, mentorship is described as an important and organically emerging outcome of the curriculum. While this is plausible and well argued, mentorship impact is inferred rather than directly measured. The authors might consider briefly noting how future iterations could incorporate explicit mentoring outcome indicators.**

**The Limitations section is appropriately concise but could be strengthened by briefly acknowledging additional potential threats to validity, such as social desirability bias, power dynamics between junior and senior faculty, or role duality (e.g., mentors also serving as evaluators or peers). Addressing these openly would enhance methodological transparency without undermining the study’s contributions.**

**The manuscript would benefit from one or two additional recent references (between 2024-2025) on “learning by doing approach” Community of practice” “peer-led or experiential faculty development” to further situate the work within current scholarship.**

**Also,  please consider briefly clarifying whether the peer review session followed a structured format (e.g., feedback prompts), as this would aid reproducibility.**

**The claim of generalisability in the Conclusion is reasonable but might be slightly softened by explicitly framing the model as transferable in principle rather than universally applicable.**

Have any limitations of the research been acknowledged?

Yes

Is the study design appropriate and does the work have academic merit?

Partly

Is the work clearly and accurately presented and does it cite the current literature?

Yes

If applicable, is the statistical analysis and its interpretation appropriate?

Yes

Are all the source data underlying the results available to ensure full reproducibility?

Partly

Are the conclusions drawn adequately supported by the results?

Yes

Are sufficient details of methods and analysis provided to allow replication by others?

Partly

Reviewer Expertise:

Faculty development, Sociolinguistics, Multiliteracies, Reflective pedagogies, Inclusive student-centred pedagogies, science education, lesson design

I confirm that I have read this submission and believe that I have an appropriate level of expertise to confirm that it is of an acceptable scientific standard, however I have significant reservations, as outlined above.
